# The role of microfibrillar‐associated protein 2 in cancer

**DOI:** 10.3389/fonc.2022.1002036

**Published:** 2022-11-30

**Authors:** Wanzhen Xu, Manfeng Wang, Yang Bai, Yong Chen, Xiaoshan Ma, Zhiqing Yang, Liyan Zhao, Yunqian Li

**Affiliations:** ^1^ Department of Neurosurgery, First Hospital of Jilin University, Changchun, China; ^2^ Department of Geriatrics, Second Affiliated Hospital of Harbin Medical University, Harbin, China; ^3^ Department of Emergency Medicine, Binzhou People’s Hospital, Binzhou, China; ^4^ Department of Clinical Laboratory, Second Hospital of Jilin University, Changchun, China

**Keywords:** MFAP2, proliferation, invasion, metastasis, extracellular matrix (ECM), apoptosis, angiogenesis, prognosis

## Abstract

Microfibrillar-associated protein 2 (MFAP2), a component of the extracellular matrix, is important in controlling growth factor signal transduction. Recent studies have shown that MFAP2, an effective prognostic molecule for various tumors, is associated with tumor occurrence and development and may be involved in remodeling the extracellular matrix and regulating proliferation, apoptosis, invasion, tumor cell metastasis, and tumor angiogenesis. However, MFAP2’s specific mechanism in these tumor processes remains unclear. This article reviewed the possible mechanism of MFAP2 in tumorigenesis and progression and provided a reference for the clinical prognosis of patients with cancer and new therapeutic target discovery.

## Introduction

The extracellular matrix (ECM) is a highly dynamic, non-cellular network composed of collagen, proteoglycans, glycosaminoglycans, elastin, fibronectin, laminins, and other glycoproteins (1, 2). ECM components are produced intracellularly by cells and released into the ECM through extracellular secretion. In normal and tumor tissues, ECM components and cell adhesion receptors bind to one another to form complex cell scaffolds for cell residence. In addition, ECM is the reservoir and binding site of bioactive molecules. Cell surface receptors transmit signals from the ECM to cells, regulating various cellular functions, including survival, growth, migration, differentiation, and immunity, for maintaining normal homeostasis (3–5). The ECM promotes cancer cell growth, survival, and invasion and modifies fibroblast and immune cell behavior to drive metastasis and impair treatment (6, 7). One study showed that metastatic liver cells promote mesenchymal cell transformation to the epithelium by regulating extracellular matrix citrullination and promoting liver metastasis progression (8). Due to its pleiotropism, the ECM can influence the fate of cells through various mechanisms (9, 10). Cells can dynamically regulate ECM remodeling in several ways, including chemical modification, composition, degradation, and reorganization (11, 12). Therefore, tumor cells interact with ECM to regulate tumor occurrence and reversal.

**Figure 1:**
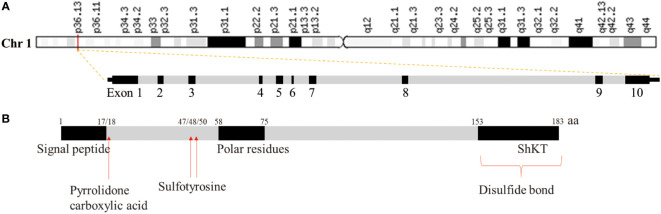
Gene **(A)** and protein **(B)** structure of MFAP2.

Microfiber-associated proteins (MFAPs) are an extracellular matrix glycoprotein group, which are extracellular matrix microfibril components involved in microfiber-assembly elastin production and tissue environmental stability (13). MFAPs include five subfamily members (*MFAP1*–*5*), among which microfibrillar-associated protein 2 (*MFAP2)* was the first to be characterized, playing an important role in controlling growth factor signal transduction (14). The human *MFAP2* gene (GenBank: GC01M016974) is located in chr1:16974502-16981583 (GRCh38/HG38), containing 10 exons with 7,082 base length and a minus-strand orientation (**Figure 1A**). Through MFAP2 transcription regulation study in muscle cells, it was found that *MFAP2* was transcribed from the main transcription start site embedded in CpG islands. A 5’flanking sequence region between nucleotides -339 and -109 is the basic *MFAP2* promoter. The -256/-270 KLF sequence motifs, an E-box at -222/-229, and a GC-box at -117/-125 are crucial for promoter function. KLF motifs mediate GKLF/KLF4 binding, whereas E-box is the target of upstream stimulatory factors 1 and 2, with the GC box forming complexes with Sp1 and Sp3 (15). The MFAP2 protein molecular structure analysis showed a signal sequence at 1–17 amino acid residues, a polar residue at 58–75, and an Shkt domain at 153–183 (**Figure 1B**), indicating that it had potential channel regulatory activity. There is a high affinity between the growth factor binding region near the MFAP2 N-terminus and the transforming growth factor-β (TGF-β) superfamily members; the mutual effect between MFAP2 and the active TGF-β form has biological consequences (16). Numerous studies have shown abnormal MFAP2 levels and its effects on prognosis in different cancers (**Table 1**), highlighting its significance in tumor progression, particularly during epithelial-mesenchymal transformation (EMT), because of its association with TGF-β (24, 31, 32). Changes in the MFAP2 level regulate ECM remodeling, playing an important role in tumorigenesis (32). In addition, MFAP2 is important in tumor cell apoptosis, proliferation, angiogenesis, invasion, and prognosis (18, 20, 23, 24).

**Table 1:** MFAP2 expression and its effects on prognosis in different types of cancers.

Type of carcinoma	Detection method or databases	Level	Expression of MFAP2	Prognosis	Key Finding	Reference
Ovarian Cancer	INTERVAL study	Protein	Upregulation	↑The risk of epithelial ovarian cancer	Represent promising new epithelial ovarian cancer biomarkers for targeted validation by studies involving direct measurement of plasma proteins in epithelial ovarian cancer patient cohorts.	(17).
	GEPIA online database	mRNA	Upregulation		MFAP2 promotes cell proliferation and glycolysis by modulating the FOXM1/β-catenin signaling pathway in ovarian cancer.	(18).
	RT-qPCR	mRNA	Upregulation			
	Western blot	Protein	Upregulation			
Colorectal adenocarcinoma	TCGA database	mRNA	Upregulation	No obvious association	MFAP2 may be involved in the development of Colorectal adenocarcinoma.	(19).
	IHC	Protein	Upregulation			
	RT-qPCR	mRNA	Upregulation			
Hepatocellular carcinoma	TCGA portal and FIREBROWSE	mRNA	Upregulation	Shorter Survival Probability, ↓OS, ↓DSS and ↓DFS	MFAP2 may be a valuable prognostic marker and an effective anticancer target, as it plays a key role in the development of hepatocellular carcinoma.	(20, 21).
	IHC	Protein	Upregulation			
	Western blot	Protein	Upregulation			
	RT-qPCR	mRNA	Upregulation			
Papillary Thyroid Cancer	RT-qPCR	mRNA	Upregulation	↓DFS, upregulation of MFAP2 was associated with higher risk of relapse or death.	MFAP2 increases the proliferation, motility and decreases the apoptosis of papillary thyroid cancer cells, and might be a potential therapeutic target for papillary thyroid cancer.	(22).
	TCGA database	mRNA	Upregulation			
Breast cancer	UALCAN and GEPIA	mRNA	Upregulation	↓OS	MFAP2 expression was increased in breast cancer tissues, and its overexpression predicted poor prognosis.	(23).
	RT-qPCR	mRNA	Upregulation			
Gastric cancer	RT-qPCR	mRNA	Upregulation	↓OS and ↓DFS	MFAP2 were identified as novel diagnostic and prognostic biomarkers in GC patients.	(24–27).
	Western blot	Protein	Upregulation			
	IHC	Protein	Upregulation			
	RNA sequencing	mRNA	Upregulation			
	GEO and TCGA databases	mRNA	Upregulation			
Head and neck squamous cell carcinoma	RT-qPCR	mRNA	Upregulation	Indicating tumor metastasis, reducing survival rate.	As a potential biomarker for prognosis and targeted therapy, MFAP2 is highly expressed in head and neck squamous cell carcinoma.	(28).
	SAGE tumor libraries	mRNA	Upregulation			
Chondrosarcoma	GEO database.	mRNA	Upregulation	↓OS and ↓DRFS	MFAP2 was poor predictor of prognosis in chondrosarcoma.	(29).
	GEPIA online database	mRNA	Upregulation			
Glioma	TCGA and CGGA databases	mRNA	Upregulation	↓OS, ↓DSS and ↓PFI	MFAP 2 is a potential prognostic marker that plays a key role in glioma development.	(30).
	Western blot	Protein	Upregulation			

DFS, Disease-free survival analysis; OS, Overall survival; DFI, Disease-free interval; DSS, Disease special survival; PFI, Progression-free interval; DRFS, Distant recurrence-free survival.

Studies on the *MFAP2* mechanism of action are expected to provide new strategies and novel therapeutic markers for cancer treatment. This review aimed to summarize *MFAP2* expression in tumor cells and its role in tumor cell proliferation, apoptosis, invasion, and metastasis and discuss its possible signaling pathways to provide new ideas for tumor therapy.

## The expression of MFAP2 in tumors

Qiu et al. (33) analyzed MFAP2 mRNA levels in different tumors and corresponding normal tissues using oncomine database. The results showed that compared with normal tissues, the expression of mfap2 was higher in bladder cancer, brain and central nervous system cancer, breast cancer, colorectal cancer, esophageal cancer, gastric cancer, head and neck cancer, lymphoma, melanoma, myeloma, ovarian cancer, pancreatic cancer and sarcoma. In order to further evaluate the expression of MFAP2 in human cancer, the integration of transcriptome data of all tumors in TCGA and GTEX showed that MFAP2 of Bladder urothelial carcinoma, Breast invasive carcinoma, Cholangiocarcinoma, Colon adenocarcinoma, Esophageal carcinoma, Head and neck squamous cell carcinoma, Liver hepatocellular carcinoma, Lung adenocarcinoma, Lung squamous cell carcinoma, Rectum adenocarcinoma, Stomach adenocarcinoma, Thyroid carcinoma and Uterine corpus endometrial carcinoma was significantly higher than that of adjacent normal tissues, and MFAP2 in Kidney chromophobe, Kidney renal clear cell carcinoma, Kidney renal papillary cell carcinoma, Prostate adenocarcinoma was significantly lower than that of adjacent normal tissues. There was no difference in MFAP2 expression in Pancreatic adenocarcinoma. In addition, the expression of MFAP2 protein increased in breast cancer, colon cancer, and lung adenocarcinoma, and decreased in clear cell renal cell carcinoma.

In addition to the *MFAP2* expression database in various tumors, extensive *in vivo* and *in vitro* experimental data have further confirmed *MFAP2* expression at the gene and protein levels in tumors. *MFAP2* expression in Bladder Urothelial Carcinoma, Breast invasive carcinoma, and Head and neck squamous cell carcinoma was detected using quantitative polymerase chain reaction (qPCR). It was found that its expression in tumor tissues was significantly higher than that in normal tissues (33). The *MFAP2* mRNA and protein levels in all ovarian cancer cell lines were much higher than those in non-tumor IOSE80 cell lines (18). In colorectal adenocarcinoma, the *MFAP2* mRNA expression in tumor tissues was significantly higher than that in the adjacent non-tumor tissues. Immunohistochemical staining has shown that the MFAP2 protein expression in tumor tissues is significantly higher than that in normal tissues (19). In hepatocellular carcinoma, quantitative real-time PCR (RT-qPCR) analysis confirmed that *MFAP2* mRNA expression was significantly upregulated in tumor tissues. Immunohistochemistry and western blotting results showed that MFAP2 protein expression in tumor tissues was significantly higher than that in adjacent normal tissues (20). Moreover, Zhu et al. (21) confirmed that upregulating *MFAP2* mRNA levels in hepatocellular carcinoma was positively correlated with the TNM stage and tumor size using RT-qPCR. In obesity-associated colon cancer, the circulating concentration of MFAP2 and its gene expression in visceral adipose tissue decreased (32). Circulating (plasma) protein levels predicted by the *MFAP2* gene were positively associated with the risk of all invasive epithelial ovarian cancers (17). In papillary thyroid carcinoma, whole-transcriptome sequencing was performed on tumor tissues and corresponding normal tissues adjacent to the carcinoma; the sequencing data were verified using RT-qPCR, confirming that *MFAP2* was highly expressed in the tumor tissue (22). In breast cancer, Gong et al. (23) found that *MFAP2* was upregulated in tumor tissues compared with that in normal tissues through bioinformatics analysis using online tools (UALCAN and GEPIA) further confirming the upregulation of *MFAP2* expression in tumor tissues and cell lines using RT-qPCR. In gastric cancer, RT-qPCR, western blotting, and immunohistochemistry analysis confirmed that *MFAP2* mRNA and protein expression levels in tumor tissues were significantly higher than those in the adjacent tissues. *MFAP2* expression level in different gastric cancer cell lines is higher than that in gastric epithelial-derived cell lines (24, 25). In head and neck squamous cell carcinoma, the *MFAP2* marker is expressed at higher levels (at least 13 times or more) in tumors than in normal tissues, as verified using RT-qPCR (28). In glioma, Our previous research shows that MFAP2 mRNA was upregulated in tumor tissues compared with that in normal tissues through bioinformatics analysis using TCGA and CGGA databases, and western blotting analysis confirmed that MFAP2 protein expression levels in tumor tissues were significantly higher than those in the adjacent tissues (30). The above data showed that *MFAP2* was upregulated in many tumor tissues, it favors specific tumor type. However, the specific mechanism needs further study.

## The carcinogenic effect of MFAP2 and its mechanisms

### The roles of MFAP2 in tumor cell proliferation

Strict proliferation regulation is critical for development; unregulated cell proliferation is an essential hallmark of cancer (34–36). A study on hepatocellular carcinoma cells showed that *MFAP2* was significantly overexpressed in hepatocellular carcinoma cells, correlating with the cancer stage. Regarding mechanism, *MFAP2* was mainly involved in ATP formation; *TP53* mutation interacted with *MFAP2* to participate in the hepatocellular carcinoma cell occurrence, and *MFAP2* knockdown inhibited hepatocellular carcinoma cell proliferation (21). *MFAP2* modulates gastric cancer cell proliferation through integrin-stimulated focal adhesion kinase activation (25). A previous study showed that *MFAP2* knockdown could inhibit breast cancer cell proliferation; *MFAP2* restoration could significantly reverse the lcpat1 knockdown effect, suggesting that *LCPAT1/MFAP2* signaling pathway may be involved in breast cancer progression (23). A papillary thyroid carcinoma study showed that *MFAP2* downregulation inhibited papillary thyroid carcinoma cell proliferation (22). *MFAP2* is a direct mir-423-5p target. mir-423-5p overexpression can downregulate MFAP2 protein expression and inhibit colon cancer cell proliferation (19). *MFAP2* overexpression significantly increased ovarian cancer cell clones’ viability and number, whereas MFAP2 knockdown produced opposite results. Similarly, *MFAP2* overexpression reduced G1 phase cell proportion and increased those of S and G2/M phase cells in ovarian cancer, suggesting that *MFAP2* could stimulate G1-S phase cell transition. In contrast, *MFAP2* knockdown induced significant arrest in the G0/G1 phase and decreased the S and G2/M phase cell proportion, indicating that cell cycle processes are affected (18). These data showed that *MFAP2* promotes tumor cell proliferation through numerous mechanisms. The inhibition of MFAP2 may inhibit the proliferation of tumor cells, and then inhibit the progress of tumor, which plays an important role in tumor treatment.

### MFAP2 is involved in tumor cell invasion and metastasis

Activating invasion and metastasis are the main cancer characteristics (34). Invasion is an initial and key step in metastasis. For invasion, tumor cells change their shape and connection with other cells and the ECM through epithelial-mesenchymal transformation (EMT). EMT is a dynamic process related to motility, invasion status acquisition, and cancer stem cell emergence (37, 38). Through EMT, tumor cells can be separated from the main tumor and invade the ECM, blood vessels, or lymphatic vessels as single cells. Therefore, EMT is involved in most tumor invasion and metastasis steps by imparting the ability to invade and spread to tumor cells. However, invasion and metastasis mechanisms vary depending on the cancer type. In addition to participating in tumor cell proliferation, MFAP2 plays an important role in tumor invasion and metastasis through EMT in tumor cells. For example, hepatocellular carcinoma cell migration and invasion ability were reduced by inhibiting EMT-related protein expression in MHCC97H cells after *MFAP2*-knockdown. Furthermore, *MFAP2* inhibition by si-*MFAP2* in YY-8103 and HuH-7 cells showed time-dependent low relative mobility (20, 21). *In vitro* colorectal adenocarcinoma studies found that *MFAP2* silencing inhibited the migration of SW480 and HCT116 cells (19). In thyroid papillary carcinoma, *MFAP2* downregulation inhibits BCPAP and TPC-1 cell migration and invasion; MFAP2 is associated with lymph node metastasis (22). *MFAP2* plays a role as an oncogene in breast cancer. After it was silenced, the MCF-7 and MDA-MB-231 cell migration and invasion abilities were impaired; after *MFAP2* was restored, the migration and invasion abilities were enhanced (23). Although in TCGA and GTEX database, there was no significant difference in the *MFAP2* expression between Skin cutaneous melanoma tumors and normal tissue, cells with *MFAP2* inhibition inhibited B16 cell invasion and migration *in vitro*. The downregulated MFAP2 cell’s ability to form tumors in the lung was significantly reduced in the nude mouse lung metastasis model established by B16 cells. These results suggest that downregulating *MFAP2* expression limits melanoma cell migration and invasion *in vitro* and *in vivo*. Moreover, *MFAP2* regulates epithelial-mesenchymal transformation to achieve this effect (39). *MFAP2* expression was upregulated in gastric cancer organization and cell lines, and its downregulation inhibited AGS and HGC-27 cell wound healing, migration, and invasion. *In vivo*, mice injected with *MFAP2* knockout cells had significantly fewer metastatic nodules on the lung and liver surfaces than those injected with control cells. Furthermore, *MFAP2*-knockdown BGC823 and MKN-45 cells significantly decreased migration and invasion by interfering with EMT (24, 25). The above studies show that MFAP2 plays a role in tumor invasion and metastasis by promoting tumor cell EMT. Since MFAP2 is an important component of extracellular matrix, more and more in-depth studies are needed to confirm whether MFAP2 can inhibit the EMT of tumor cells and inhibit the invasion and metastasis of tumor cells in all tumors.

### MFAP2 inhibits tumor cell apoptosis

As a complex regulator, apoptosis plays an important role in maintaining development and homeostasis (40). The apoptosis mechanism imbalance is associated with various diseases; the most concerning is carcinogenesis and malignant tumor occurrence and development (34). Apoptosis is a distinguishing cancer feature, and its deregulation often leads to chemoresistance (41). Therefore, the therapeutic method of specifically inducing cancer cell apoptosis has undeniable value in the fight against this type of disease. Significant efforts have also been made in developing and researching related drugs (42, 43). Dong et al. showed that *MFAP2*, an oncogene in thyroid papillary carcinoma, was overexpressed in thyroid papillary carcinoma compared to normal tissues. The *MFAP2* expression level was significantly correlated with lymph node metastasis, tissue type, and tumor focus type, and its downregulation induced BCPAP and TPC-1 cell apoptosis. Therefore, *MFAP2* downregulation promotes apoptosis in thyroid papillary carcinoma cells (22). As an oncogene in breast cancer, MFAP2 knockout can significantly increase MCF-7 and MDA-MB-231 cell apoptosis. However, restoring *MFAP2* expression can reduce apoptosis. Moreover, lncRNA LCPAT1 interacts with RBBP4 and recruits it to the *MFAP2* promoter, promoting breast cancer progression by activating MFAP2 transcription (23). In colon adenocarcinoma, silencing *MFAP2* promoted SW480 and HCT116 cell apoptosis. The increased apoptosis induced by si-kcnq1ot1 and mir-423-5p simulated transfection could be neutralized by transferring the *MFAP2* overexpression plasmid into SW480 and HCT116 cells. The results showed that MFAP2 inhibits tumor cell apoptosis in colon adenocarcinoma (19). The above results indicate that MFAP2 can inhibit the apoptosis of tumor cells and promote the survival of tumor cells through its high expression in tumor cells. Targeting MFAP2 may play an important role in tumor therapy.

### The roles of MFAP2 in tumor angiogenesis

The circulatory system is essential for delivering nutrients and chemicals to tissues, cleaning waste, and maintaining homeostasis. Malignant tumor cells require oxygen and nutrients for survival and reproduction (44, 45). Continuous angiogenesis is a hallmark of cancer. In addition to providing oxygen and nutrition, newly formed blood vessels also produce paracrine factors to support the cancer microenvironment and promote tumor growth, progression, and metastasis (46–50). However, although the current anti-angiogenic drugs initially had a breakthrough, they encountered major difficulties in follow-up trials and failed to be used in treating most malignant solid tumors, possibly due to low clearance efficiency or insufficient inhibition of tumor-related endothelial cell functions (51–53). During blood vessel development, endothelial cells exit their quiescent state, migrate, and proliferate into the surrounding and previously degraded matrix in response to angiogenic stimuli to form new functional blood vessels (54–56). Therefore, developing new therapies that inhibit endothelial cells is essential in cancer treatment (57, 58). Epidermal growth factor-like protein-7 (EGFL7) is involved in blood vessel development, and vascular lumen formation drives tumor angiogenesis, contributing to the pathological tumor vascular phenotype (59, 60); these EGFL7 features contribute to blood vessel wall instability and promote vascular leakage, characteristic of tumor endothelial cells (61). EGFL7 promotes glioma growth and stimulates tumor vascularization by generating mature blood vessels covered by pericytes and smooth muscle cells (62). High EGFL7 levels are associated with higher tumor grade and poor prognosis as a potential cancer target. However, it was found that MFAP2 is necessary for depositing EGFL7 into microfibrils, which is crucial for vascular development, showing that MFAP2 is important in tumor angiogenesis (63). An *in vitro* human umbilical vein endothelial cells (HUVEC) tube-forming experiment showed that vascular endothelial growth factor A decreased significantly after *MFAP2* knockdown in hepatocellular carcinoma. The *MFAP2* knockout MHCC97H cell supernatant reduced HUVEC’s average tube length, network, and branch number. These results showed that silencing *MFAP2* could reduce angiogenesis (20). These findings emphasize the important role of MFAP2 in tumor hematoma formation. Considering the importance of neovascularization for tumor progression, targeted MFAP2 therapy may play an active role in tumor therapy, especially for vascular rich tumors. Therefore, further studies are needed to reveal *MFAP2’s* role and mechanism in tumor angiogenesis.

### Related signal pathways of MFAP2 in tumors

**Figure 2:**
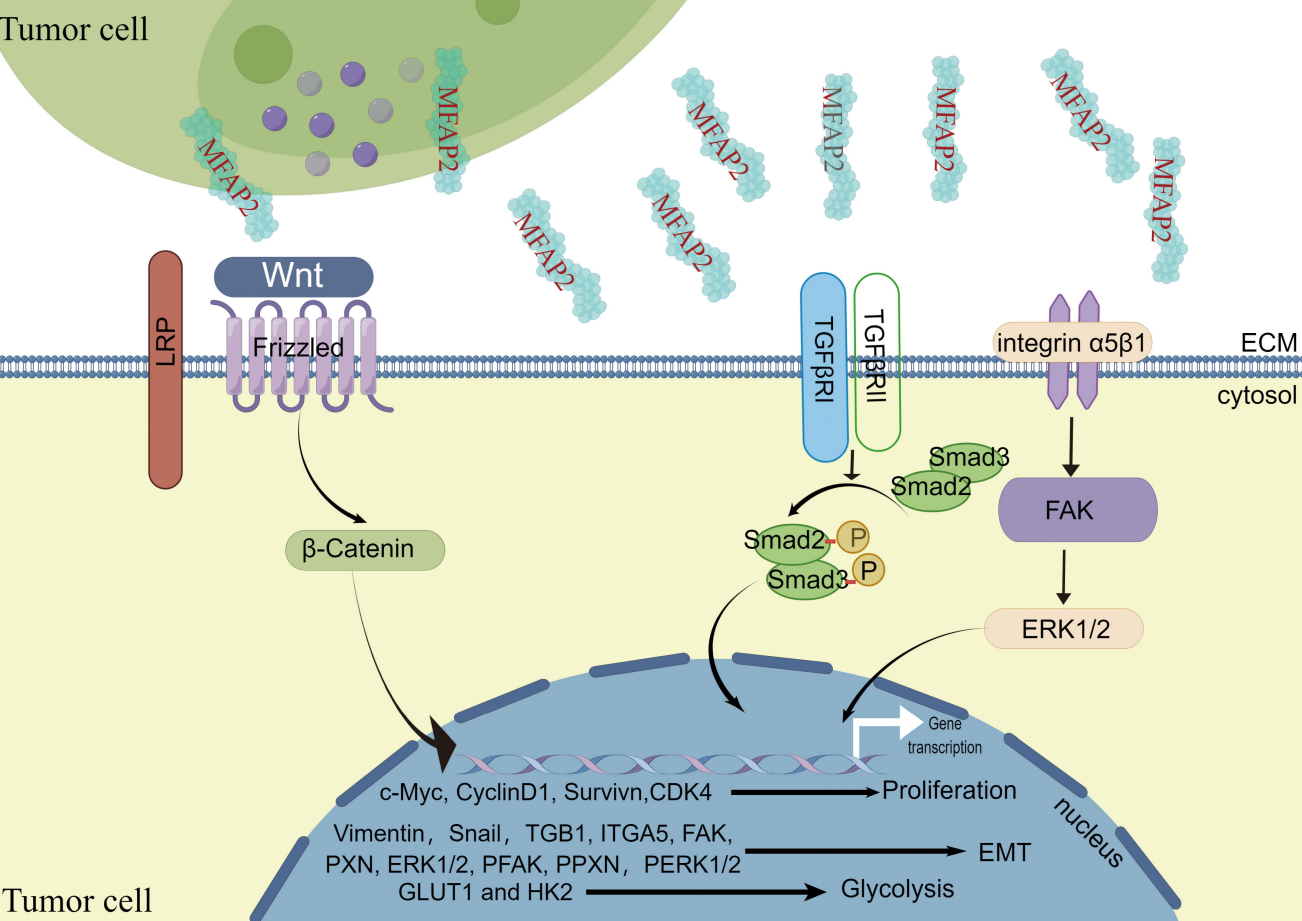
The carcinogenic effect of MFAP2 and its mechanism.

Cancer is characterized by genetic changes that affect signaling pathways controlling cell cycle progression, apoptosis, and cell growth; however, these changes’ extent, mechanisms, and co-occurrence vary (64). Various authors have shown that MFAP2 overexpression promotes tumor progression by activating cellular signaling pathways (**Figure 2**).

***Wnt signaling pathway.*** Although the mechanism of the Wnt signaling pathway varies in different physiological processes, its signal transduction dysregulation can contribute to human diseases (65). Current methods for Wnt signaling mainly target cancer interventions. The abnormal Wnt signaling pathway is involved in various tumors, including pancreatic, lung, breast, ovarian, and colorectal cancers (66, 67). Wnt/β-catenin is one of the main signaling pathways contributing to EMT in various cancer growth and progression (68, 69). In melanoma, MFAP2 disruption influenced Wnt/β-catenin linked protein levels, and β-catenin neutralized MFAP2’s effect on EMT, as evidenced by the altered EMT molecular markers’ protein levels. This study showed that MFAP2 activates the Wnt/β-catenin signaling pathway and enhances EMT during melanoma metastasis (39).Forkhead box M1 (FoxM1) belongs to the forkhead box transcription factor family. It is abnormally expressed in various human cancers and plays a role in tumor initiation, progression, invasion, metastasis, and angiogenesis (70). Studies have shown that FOXM1 strongly enhances cancer cellβ-catenin nuclear translocation (71, 72). Signaling activation relies on β-catenin nuclear localization, and FOXM1 promotes it in different cancer cells, which is highly correlated with tumor metastasis (71, 73, 74). In contrast, cancer cell energy requirements are increased, and metabolic reprogramming, a cancer cell marker, is critical for tumor growth and metastasis (75). In ovarian cancer, MFAP2 promotes β-catenin transfer from the cytoplasm to the nucleus *via* the FoxM1/β-Catenin signal pathway, increasing the glycolysis-related gene (GLUT1 and HK2) expression levels in A2780 and SKOV3 cells (18).

***Integrin/FAK/ERK1/2 signaling pathway.*** Increased integrin expression leads to focal adhesion kinase (FAK) activation, activating survival pathways, including PI3K/Akt, and promoting cell migration and invasion (76–78). It has been reported that in gastric cancer, MFAP2 plays an important role in regulating the integrin signaling pathway in tumor cell ECM interaction as an integrin/FAK/ERK1/2 signaling activator during gastric cancer progression (25).

***TGF-β signaling pathway.*** The TGF-β signaling pathway is involved in oncogenesis in multiple ways (79, 80). Moreover, TGF-β acts as a tumor inhibitor in the early oncogenesis phases; however, it may also enhance tumor progression in later phases (81). The classic SMAD pathway involves binding TGF-β receptors TβRI and TβRII to their respective ligands, forming a tetramer compound containing phosphorylated Smad2 and Smad3. After translocating to the nucleus, phosphorylated Smad2 and Smad3 interact with Smad4 to regulate epithelial-mesenchymal transition. Epithelial cells acquire mesenchymal characteristics because of epithelial-mesenchymal transition, resulting in increased migration, invasiveness, and resistance to therapies (82, 83). It has been reported in gastric cancer that MFAP2 knockdown significantly reduced TGF-β expression and activity, as assessed by Smad-2 and Smad-3 phosphorylation. However, TGF-β treatment reversed MFAP2 knockdown gastric cancer cell damaged migration and invasion without changing MFAP2 expression. Therefore, MFAP2 acts as an upstream regulator by activating TGF-β/Smad2/3 signaling, promoting proliferation, migration, invasion, and EMT phenotype (24). Similarly, the biological analysis showed that MFAP2 combines with TGF-β and BMP through its highly acidic sequence near the N-terminus. MFAP2 binds to active TGF‐β1 but not to latent TGF‐β1. *MFAP2* deletion increases the total TGF-β stored in cultured cells’ ECM because the active TGF‐β1 does not bind to fibrillin, indicating that MFAP2 plays an active role in TGFβ signaling in the ECM (31).

The above research results show that MFAP2 participates in multiple signal pathways and plays an important role in tumor invasion, metastasis, metabolism and resistance to therapies in tumors, emphasizing that MFAP2 may be used as a major target of anti-tumor therapy to develop relevant drugs.

## Prognostic value of MFAP2 in tumors

Identifying the molecular mechanisms responsible for tumor development and maintenance is essential in developing targeted cancer therapies (84). This can be achieved by compiling genetic alterations across multiple tumor types in large-scale cross-sectional molecular studies for cancer, including The Cancer Genome Atlas (TCGA) and the International Cancer Genome Consortium (85, 86). Some authors have shown that MFAP2 can be a prognostic indicator for various tumors. Through the pan cancer analysis of MFAP2, Qiu et al. (33) showed that high expression of MFAP2 was associated with poor DFI for Adrenocortical carcinoma, Breast invasive carcinoma, Cervical squamous cell carcinoma and endocervical adenocarcinoma, Cholangiocarcinoma, Ovarian serous cystadenocarcinoma, and Pancreatic adenocarcinoma, with poor DSS for Adrenocortical carcinoma, Breast invasive carcinoma, Cervical squamous cell carcinoma and endocervical adenocarcinoma, Cholangiocarcinoma, Kidney renal clear cell carcinoma, Brain lower grade glioma, Liver hepatocellular carcinoma, and Sarcoma, with poor OS for Adrenocortical carcinoma, Breast invasive carcinoma, Cervical squamous cell carcinoma and endocervical adenocarcinoma, Kidney renal clear cell carcinoma, Brain lower grade glioma, Liver hepatocellular carcinoma, and Sarcoma, and poor PFI for Adrenocortical carcinoma, Bladder urothelial carcinoma, Breast invasive carcinoma, Cervical squamous cell carcinoma and endocervical adenocarcinoma, Kidney chromophobe, Kidney renal clear cell carcinoma, Brain lower grade glioma, and Sarcoma. In the specific analysis of each cancer, as a pivotal gene related to tumorigenesis, *MFAP2* is a biomarker for the diagnosis and prognosis of gastric cancer. It contributes to personalized treatment and is an independent prognostic factor for the overall survival of patients with gastric cancer. And in gastric adenocarcinoma, high MFAP2 expression was significantly correlated with the first progression and post-progressive survival shortening; the overall and disease-free survivals were shorter in patients with increased MFAP2 expression (26, 27). Studies have shown that *MFAP2* is significantly upregulated in hepatocellular carcinoma and is associated with tumor progression and prognosis. The higher the MFAP2 expression, the worse the prognosis and the lower the survival rate. MFAP2 promotes hepatocellular carcinoma cell proliferation, migration, and invasion. MFAP2 may also be a promising prognostic biomarker with important clinical significance as a potential immunotherapeutic target for hepatocellular carcinoma patients (21). *MFAP2* is a poor prognosis marker in chondrosarcoma malignant transformation; its high expression predicts poor prognosis (29). The predicted circulating MFAP2 protein level is associated with epithelial ovarian cancer risk. The regional genome map confirms the genetic association signal overlap between the plasma protein level and the risk of epithelial ovarian cancer (17). Studies have shown that *MFAP2* expression can distinguish papillary thyroid cancer tissues from normal ones. Upregulated *MFAP2* expression in papillary thyroid cancer is associated with an increased recurrence or death risk. Therefore, *MFAP2* overexpression predicts a poor prognosis in papillary thyroid cancer (22). As a potential biomarker of head and neck squamous cell carcinoma tumorigenesis, *MFAP2* is significantly overexpressed in SAGE tumor libraries (28). In addition, *MFAP2* is a prognostic marker that correlates with the immune microenvironment in glioma (30). MFAP2 is important in the prognosis of the above tumor types; however, the specific mechanism and prognostic value of MFAP2 in other cancers with low expression of MFAP2, such as Kidney chromophore, Kidney renal clear cell carcinoma, Kidney renal papillary cell carcinoma, and Prostate adenocarcinoma, need to be further explored.

## Relationship between MFAP2 expression level and immune infiltration in tumors

The interaction of various factors constituting the tumor microenvironment forms the characteristics of cancer and has a significant impact on the antitumor immune response (34). These factors include cell components, surrounding extracellular matrix and interstitial fluid. Among them, the cellular components include tumor cells themselves and stromal cells such as fibroblasts, endothelial cells and infiltrating immune cells. As part of the immune response against cancer, infiltrating immune cells play a crucial role (87). Zhu X et al. investigated the relationship between the expression of *MFAP2* and immune factors in Hepatocellular carcinoma and got the fact that some immunostimulators, immunoinhibitors, and Regulatory T cells for which expression was significantly correlated with MFAP2 expression by filtering (21). Qiu Z et al. analyzed the interaction of MFAP2 with various immune cell infiltration, and found that the expression of *MFAP2* was significantly positively correlated with the infiltration level of B cells, CD4+T cells, CD8+T cells, dendritic cells, macrophages and neutrophils in bladder urothelial carcinoma, breast invasive carcinoma and low-grade glioma. In addition, MFAP2 might regulate macrophage polarization in Bladder Urothelial Carcinoma, Colon adenocarcinoma, Esophageal carcinoma, Kidney Chromophobe, Brain Lower Grade Glioma, Liver hepatocellular carcinoma, Pancreatic adenocarcinoma, Prostate adenocarcinoma, Rectum adenocarcinoma, Stomach adenocarcinoma, Head and Neck squamous cell carcinoma-HPV-, Thyroid carcinoma, and Thymoma (33). In our previous research, the expression level of *MFAP2* in glioma was positively correlated with Th2 cells, macrophages, eosinophils, neutrophils and T cells. Additionally, *MFAP2* expression level in glioma was positively correlated with key markers of T-cell exhaustion (30). These results suggest that the high expression of MFAP2 in different types of tumors can form tumor immune infiltration, and then regulate tumor microenvironment, which plays an important role in immunotherapy of a variety of tumors, deserve further studies for their potential clinical implications.

## Discussion

MFAP2 is known for its function in microfibril assembly (88). It has been shown that *MFAP2* ^(−/−)^ mice exhibit obesity, metabolic dysfunction, adipocyte hypertrophy, and reduced heat generation. TGF-β activity increased in *MFAP2* ^(−/−)^ adipose tissue, and treating *MFAP2* ^(−/−)^ mice with a TGF-β-neutralizing antibody improved their body temperature and prevented the increased obesity (89). In addition, MFAP2 is a bone remodeling regulator; its deficient mice show progressive osteopenia, accompanied by increased osteoclasts and NF- κB ligand-receptor activator expression (90, 91). MFAP2 expression is diverse at different developmental stages, highest in fetuses and newborns and lowest in adults (92). In addition, by summarizing MFAP2 research, it was found that its protein is not necessary for normal development; however, mutations in the MFAP2 gene cause defects in multiple organ systems (14). Recently, it was found that MFAP2 affects different tissue tropisms, playing an important role in regulating steady tissue state, cell survival, and tumor progression (13). Bioinformatics and computational biology play critical roles in bioscience and biomedical research (93). Therefore, *MFAP2* expression in tumor tissues and its prognostic relevance were determined. Targeting *MFAP2* is important in inhibiting tumor cell proliferation, migration, invasion, angiogenesis, and promoting tumor cell apoptosis. Similar conclusions were verified in *in vivo* and *in vitro* experiments. Regarding the mechanism, *MFAP2* exerts oncogenic function through an alternative mechanism because it is associated with microfibrils in the ECM and induces other matrix remodeling gene expressions, including Versican (94). *MFAP2* dysregulation greatly changes the ECM status in the cancer microenvironment, modulating the cancer cell phenotypes (25). Therefore, MFAP2-targeted therapy may play an important role in adult tumor therapy with minimal collateral damage. Zhu et al. (13) provided us with a comprehensive understanding of microfibril related proteins in diseases by summarizing the molecular structure and function of microfibril related proteins in bone, metabolic disorders, and cancer. In light of novel data on microfibril-related proteins, including the role and mechanism of MFAP2 in tumors, we focused on the role and mechanism of MFAP2 in cancer to provide an updated review. However, insights have been restricted to studies employing siRNA-mediated knockdown of expression or the generation of knockout lines of cell and mice until recently. Despite these approaches, the specific functions of MFAP2 in cancer remain uncertain, and this possibility must be addressed experimentally with specific pharmacological tools. Additionally, the study on the regulation mechanism upstream of MFAP2 shows that the expression of MFAP2 is regulated by lncRNAs, such as lncRNA LCPAT1 (23) and lncRNA KCNQ1OT1 (19). To compare MFAP2 expression and degradation in tumor versus healthy cells, and aid anti-tumor drug development, it is necessary to conduct an in-depth study on the regulation mechanism of MFAP2 in tumors.

In conclusion, the MFAP2 mechanism in various cancers requires further exploration. Identifying and utilizing MFAP2 involvement in cancer will help develop new diagnostic, therapeutic, and prognostic methods for patients with malignant tumors.

## Author contributions

WX, MW and YB designed and analyzed the research. WX and MW drafted the manuscript. LZ and YL participated in the critical revision of the manuscript. XM, YC and ZY participated in the data collection and literal modification of the manuscript. All authors read and approved the final manuscript.

## Funding

This work was supported by National Nature and Science Foundation of China (81672505), the S&T Development Planning Program of Jilin Province (20200404101YY and 20200201613JC), Jilin Province Medical and Health Talent Project (JLSWSRCZX2021-052) and Health and Wellness Technology Enhancement Project of Jilin Province (2021LC007).

## Acknowledgments

**Figure 2** was drawn by Figdraw; and we would like to thank Editage (www.editage.cn) for English language editing.

## Conflict of interest

The authors declare that the research was conducted in the absence of any commercial or financial relationships that could be construed as a potential conflict of interest.

## Publisher’s note

All claims expressed in this article are solely those of the authors and do not necessarily represent those of their affiliated organizations, or those of the publisher, the editors and the reviewers. Any product that may be evaluated in this article, or claim that may be made by its manufacturer, is not guaranteed or endorsed by the publisher.
